# Reduced B cell frequencies in cord blood of HIV-exposed uninfected infants: an immunological and transcriptomic analysis

**DOI:** 10.3389/fimmu.2024.1445239

**Published:** 2024-09-04

**Authors:** Ye Jin, Jun Zhao, Tangkai Qi, Di Tian, Yixin Liao, Qing Yang, Minming Li, Qingqing Zhu, Jun Chen, Yinzhong Shen, Yabin Liu, Hongzhou Lu

**Affiliations:** ^1^ Department of Infection Immunology, Shanghai Public Health Clinical Center, Fudan University, Shanghai, China; ^2^ Children's Health Department, Shanghai Center for Women and Children’s Health, Shanghai, China; ^3^ Department of Pediatrics, Shanghai Public Health Clinical Center, Fudan University, Shanghai, China; ^4^ Scientific Research Center, Shanghai Public Health Clinical Center, Fudan University, Shanghai, China; ^5^ Child Healthcare Department, Songjiang Maternity and Child Health Hospital, Shanghai, China; ^6^ National Clinical Research Center for Infectious Diseases, Third People’s Hospital of Shenzhen, Second Affiliated Hospital of Southern University of Science and Technology, Shenzhen, China

**Keywords:** HIV-exposed uninfected, cord blood, cell counting, B lymphocyte, transcriptome, immune system

## Abstract

**Introduction:**

In the course of immune development, HIV-exposed uninfected (HEU) infants exhibit abnormal immune function and increased infectious morbidity compared to HIV-unexposed uninfected (HUU) infants. Yet the specific functional phenotypes and regulatory mechanisms associated with *in-utero* HIV and/or ART exposure remain largely obscure.

**Methods:**

We utilized flow cytometry and RNA-seq technologies to conduct the immunological and transcriptomic profiling in cord blood from 9 HEU mother-infant pairs and 24 HUU pairs. On top of that, we compared the cord blood dataset with the maternal venous blood dataset to characterize unique effects induced by *in-utero* HIV and/or ART exposure.

**Results:**

Flow cytometry immunophenotyping revealed that the level of B lymphocyte subsets was significantly decreased in HEU cord blood as compared to HUU (*P* < 0.001). Expression profiling-based cell abundance assessment, includes CIBERSORT and ssGSEA algorithm, showed a significantly reduced abundance of naive B cells in HEU cord blood (both *P* < 0.05), supporting the altered composition of B lymphocyte subsets in HEU. Functional enrichment analysis demonstrated suppressed innate immune responses and impaired immune regulatory function of B cells in HEU cord blood. Furthermore, through differential expression analysis, co-expression network analysis using WGCNA, and feature selection analysis using LASSO, we identified a 4-gene signature associated with HEU status. This signature effectively assesses B cell levels in cord blood, enabling discrimination between HEU and HUU infants.

**Discussion:**

Our study provides the first comprehensive immunological and transcriptomic characterization of HEU cord blood. Additionally, we establish a 4-gene-based classifier that holds potential for predict immunological abnormalities in HEU infants.

## 1 Introduction

Despite the efficacy of anti-retroviral therapy (ART) in preventing human immunodeficiency virus (HIV) transmission from mother to child, *in-utero* HIV-exposed uninfected (HEU) infants experience significantly higher morbidity and mortality compared to HIV-unexposed uninfected (HUU) infants (1). Particularly within the first 12 months of life, HEU infants exhibit increased hospitalization rates due to increased susceptibility to common childhood pathogens, including *respiratory syncytial virus* (RSV), *Haemophilus influenza*, and *Streptococcus pneumoniae* (2–7). Factors such as maternal HIV infection, antiretroviral drugs, vaccination, and gut microbiome have been implicated in the impaired clinical outcomes of HEU infants (8–13), affecting their immune system development and increasing their susceptibility to infections. However, the mechanisms by which *in-utero* HIV and/or ART exposure contribute to these vulnerabilities remain poorly understood, necessitating systematic exploration.

The preponderance of current evidence demonstrates that the immune system of HEU infants, encompassing both innate and adaptive immunity, differs from that of HUU infants (14). HEU infants have lower levels of maternal antibodies at birth (15–17), significant defects in T cell functionality (18), heightened monocyte activation (19), and increased NK cell cytotoxicity (20), which persist beyond the first year of life. However, the relationship between different immune cell dysfunctions and the underlying mechanisms has been relatively poorly elucidated. Systematic immunophenotypic and transcriptomic profiling of HEU infants at birth can expand our understanding of the immunologic impact of *in-utero* HIV and/or ART exposure.

Previous studies have shown that HEU cord blood exhibits lower antibody levels, distinct cytokine profiles, and reduced T cell helper responses compared to HUU cord blood (21–23), suggesting that immunological abnormalities in cord blood correlate with immune alterations observed at birth induced by *in-utero* HIV and/or ART exposure. In this regard, analyzing biomarkers in cord blood may be useful for estimating the degree of immune system abnormality in HEU infants, as cord blood can directly reflect fetal status, including infection and inflammation.

Here, we utilized flow cytometry and RNA-seq technologies to establish the immune and transcriptional profiles in cord blood from 9 HEU and 24 HUU mother-infant pairs. Comparative analyses of the cord blood and maternal venous blood datasets were conducted at both the immunophenotypic and transcriptomic levels. CIBERSORT and ssGSEA were employed to evaluate differences in immune cell abundance between HEU and HUU, corroborating the altered immune cell levels measured by flow cytometry. Differential expression and functional enrichment analyses were utilized to investigate the mechanisms underlying immunologic dysfunctions induced by *in-utero* HIV and/or ART exposure. Integration and analysis of both datasets using WGCNA and LASSO identified HEU-related signatures. Our findings provide highlight altered B lymphocyte composition in cord blood as an indicator of immunologic dysfunction in HEU infants. We also developed a 4-gene-based classifier for predicting the immunological abnormalities in HEU infants at birth, underscoring the significant role of B lymphocyte function in their abnormal immune development.

## 2 Materials and methods

### 2.1 Patient recruitment and ethical approval

This study was conducted in Shanghai, China by the Shanghai Public Health Clinical Center and Shanghai Songjiang Maternal and Child Health Hospital. The Shanghai Public Health Clinical Center Ethics Committee approved this study. Written informed consent was obtained from all participants. Nine HEU mother-infant pairs and 24 HUU mother-infant pairs were included, enrolled from 2020 to 2021 (**Supplementary Tables 1**, **2**). All HIV-infected mothers received antiretroviral treatment. Maternal venous blood, and infant umbilical cord blood were collected during delivery.

### 2.2 Flow cytometry analysis

2 ml of venous blood or cord blood were collected and transferred into an EDTA anticoagulant tube (purple tube). Antibodies (Beckman Coulter) were added to the whole blood or cord blood sample for testing. The samples were incubated for 15 to 20 minutes at room temperature (18 – 25°C), protected from light. Red cell lysis was performed, followed by centrifugation and washing of the cells using PBS twice. The supernatant was removed, and the cell pellets were resuspended using PBS. The preparations were analyzed within 2 hours using a Beckman Coulter flow cytometer (Navios) equipped with three lasers and ten colors. Data analysis was performed using Kaluza software. Antibodies for flow cytometry were listed in **Supplementary Table 3**. Subsequent data analysis was conducted using specialized software to gate and quantify the populations of interest (details in **Supplementary Methods**).

### 2.3 RNA-seq library construction

2.5mL of venous blood and cord blood were collected and injected it into a BD PAXgene blood RNA tube. The tube was mixed by inversion. Total RNA was extracted using the RNeasy mini kit (QIAGEN) according to the manufacturer’s instructions. 1 μg of total RNA was used for library preparation. RNA-seq libraries were constructed using the TruSeq RNA Sample Preparation Kit v2 (Cat. # RS-122-2001 or RS-122-2002; Illumina, Hayward, CA, USA) according to the manufacturer’s instruction. The purified library was quantified using Qubit3.0 Fluorometer (Invitrogen, Carlsbad, CA, USA), and size distribution was analyzed by Bioanalyzer 2100 (Agilent Technologies, Palo Alto, CA). High throughput sequencing was performed on the Illumina HiSeq 2500 or NovaSeq 6000 platform.

### 2.4 Statistical analysis

All statistical analyses and plots were conducted using R (v4.3.0). The Wilcoxon rank-sum test was employed to compare groups with non-normally distributed variables, as this non-parametric test dose not assume normal distribution and is appropriate for small sample sizes. LASSO (Least Absolute Shrinkage and Selection Operator) feature selection analysis was utilized to identify HEU-associated signatures. LASSO was chosen because it effectively handles high-dimensional data and performs both variable selection and regularization to enhance the prediction accuracy and interpretability of the statistical model. This analysis was conducted using the R package Glmnet (v4.1-7). Correlation coefficients were assessed by Spearman analysis, chosen for its ability to measure the strength and direction of association between two ranked variables without assuming a linear relationship or normal distribution. Statistical significance was defined as *P* < 0.05.

For a detailed description of the data analysis methods, see **Supplementary Methods**.

## 3 Results

### 3.1 Immune profiling reveals a significant decrease in B cell levels in HEU cord blood

HEU children exhibit a potentially compromised immune system compared to HUU children, rendering them more susceptible to pathogens during their growth and development. Thus, we propose that optimizing infant immunization schedules warrants exploration and understanding of the immunological abnormalities observed in HEU newborns.

#### 3.1.1 Study population and sample collection

**Figure 1:**
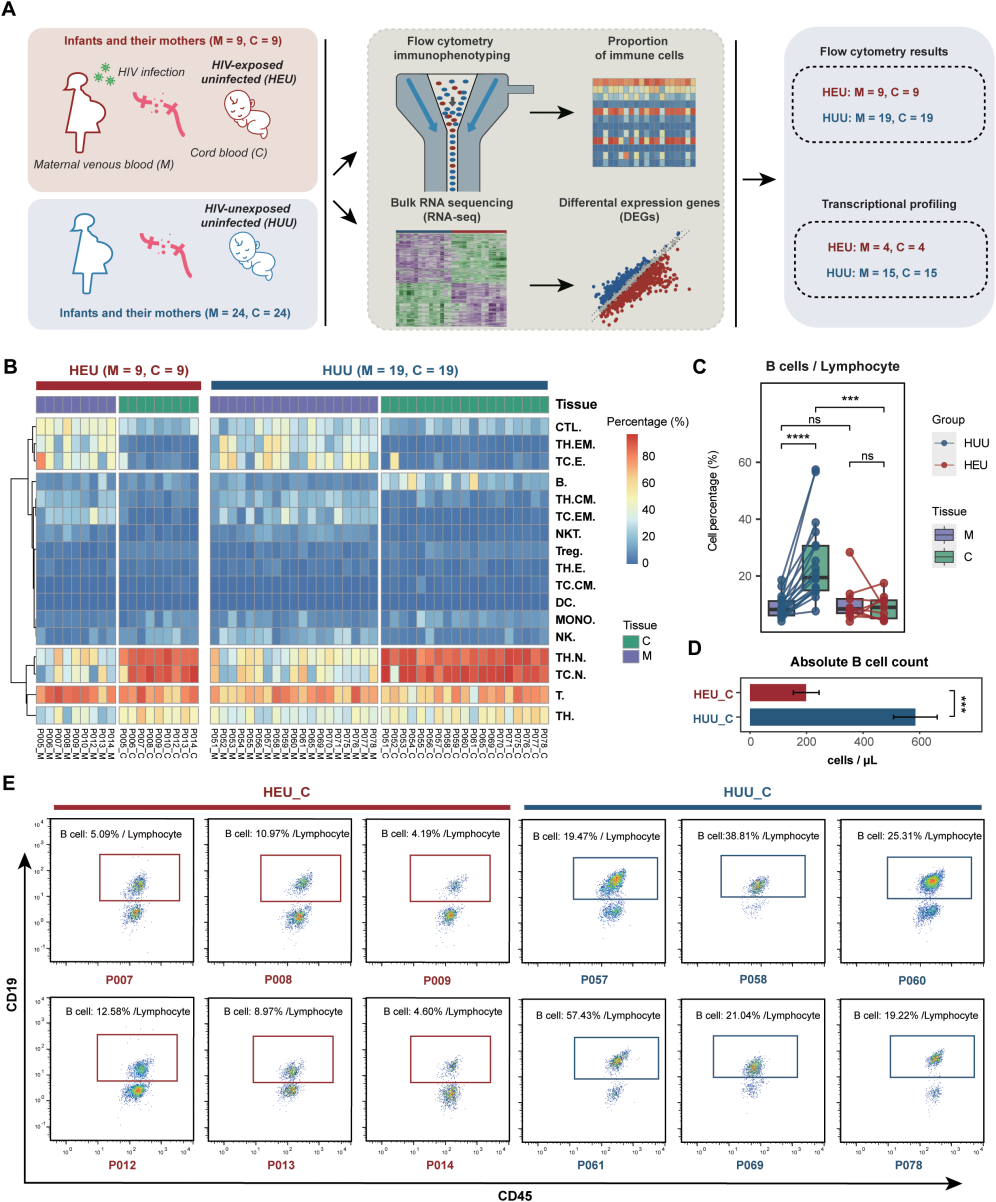
Comparison of immune profiles between HEU and HUU. **(A)** Schematic diagram illustrating the experimental design for flow cytometry immunophenotyping and bulk RNA sequencing. Paired samples, including maternal venous blood (abbreviated as M) and umbilical cord blood (abbreviated as C), were collected from HEU infants and their mothers. Paired maternal venous blood and umbilical cord blood samples from HUU mother-infant pairs served as controls for this experiment. **(B)** Heatmap displaying the immune profiles measured by flow cytometry in the HEU and HUU subjects. **(C)** Matched analysis of the compositions of B cells in maternal venous blood, and umbilical cord blood samples across the HEU and HUU subjects. The ordinate represents the percentage of cells in the lymphocyte. Each box plot represents the median, interquartile range, and minimum and maximum quartile of expression. The *P* value was calculated by the Wilcoxon test. ***P < 0.001, ****P < 0.0001; ns, not significant. **(D)** Comparison of the absolute B cell counts in cord blood samples between HEU and HUU groups. Absolute B cell counts were determined using a Beckman Coulter flow cytometer. Counting beads were added to the samples for quantification. Single cells were gated based on forward scatter (FSC) and side scatter (SSC) to exclude debris and aggregates. Lymphocytes were gated using SSC and CD45 expression, and B cells were identified by the CD19 surface marker. The absolute B cell number was calculated using the ratio of CD19+ B cells to counting beads, ensuring accurate counts in the cord blood samples. Data are plotted as means plus or minus standard deviation. ***P < 0.001. **(E)** Representative flow cytometry analysis of B lymphocytes (CD3-CD19+) in cord blood samples from HEU and HUU subjects. The data depict CD19-positive cells gated on CD45+CD3- events. The frequency of B lymphocytes, expressed as a percentage of all lymphocytes (gated on CD45 vs. SSC), is shown.

To systematically characterize immune differences between HEU and HUU infants at birth, we established the immune and transcriptional profiles in matched cord blood and venous blood samples from 9 mother-infant pairs from HIV+ mothers and 24 HIV-uninfected mothers (**Figure 1A**; **Supplementary Table 2**). Both maternal groups were of similar time of gestation (**Supplementary Figure 1A**). The sex distribution of HEU and HUU infants was also comparable. At birth, HEU infants demonstrated relatively low weight, with a mean of 3303g compared to 3401g in HUU infants, although statistical significance was not reached due to sample size limitations (**Supplementary Figure 1B**).

#### 3.1.2 Comparative analysis of immune cell composition

The composition of various immune cell subsets was measured using flow cytometry (**Supplementary Tables 3**, **4**; **Supplementary Figure 2**; details in **Supplementary Methods**). Notably, significant variations in immune cell composition were evident between maternal venous blood and umbilical cord blood across both HEU and HUU groups (**Figure 1B**), suggesting that immunological abnormalities in cord blood might indicate the altered immune system status of corresponding infants. Furthermore, we performed a comparative analysis of immune cell composition from different sample sources (**Supplementary Figure 3**). As expected, due to HIV infection and antiretroviral therapy, statistically significant differences were observed across multiple immune cell subsets (including CTL, DC, MONO, NK, and T cells) between venous blood samples from HIV-positive mothers and HIV-uninfected mothers.

#### 3.1.3 B cell levels in HEU cord blood

Interestingly, we observed significantly reduced B cell levels in HEU cord blood compared to HUU cord blood samples. In the HUU group, cord blood B cell levels were significantly lower than the maternal venous blood B cell levels, whereas in the HEU group, there was no difference between cord blood and maternal venous blood B cell levels (**Figure 1C**). Additionally, a comparison of the absolute B cell values in cord blood revealed that the HEU group (198.619 ± 45.616) had significantly lower values than the HUU group (584.996 ± 77.176) (*P* < 0.001) (**Figure 1D**). Representative flow cytometry analysis in **Figure 1E** further illustrates that the proportion of B cells within the lymphocyte population is considerably smaller in HEU cord blood compared to HUU cord blood. These data suggested that diminished B cell levels in HEU cord blood are linked to immunological abnormalities in HEU infants, possibly stemming from *in-utero* HIV and/or ART exposure. Consequently, the findings indicate that *in-utero* HIV and/or ART exposure appeared to lead to notable reductions in B cell levels, potentially contributing to B cell dysfunction during immune development in HEU infants.

### 3.2 Inferring alterations in the composition of B lymphocyte subsets in cord blood via transcriptional profiling

**Figure 2:**
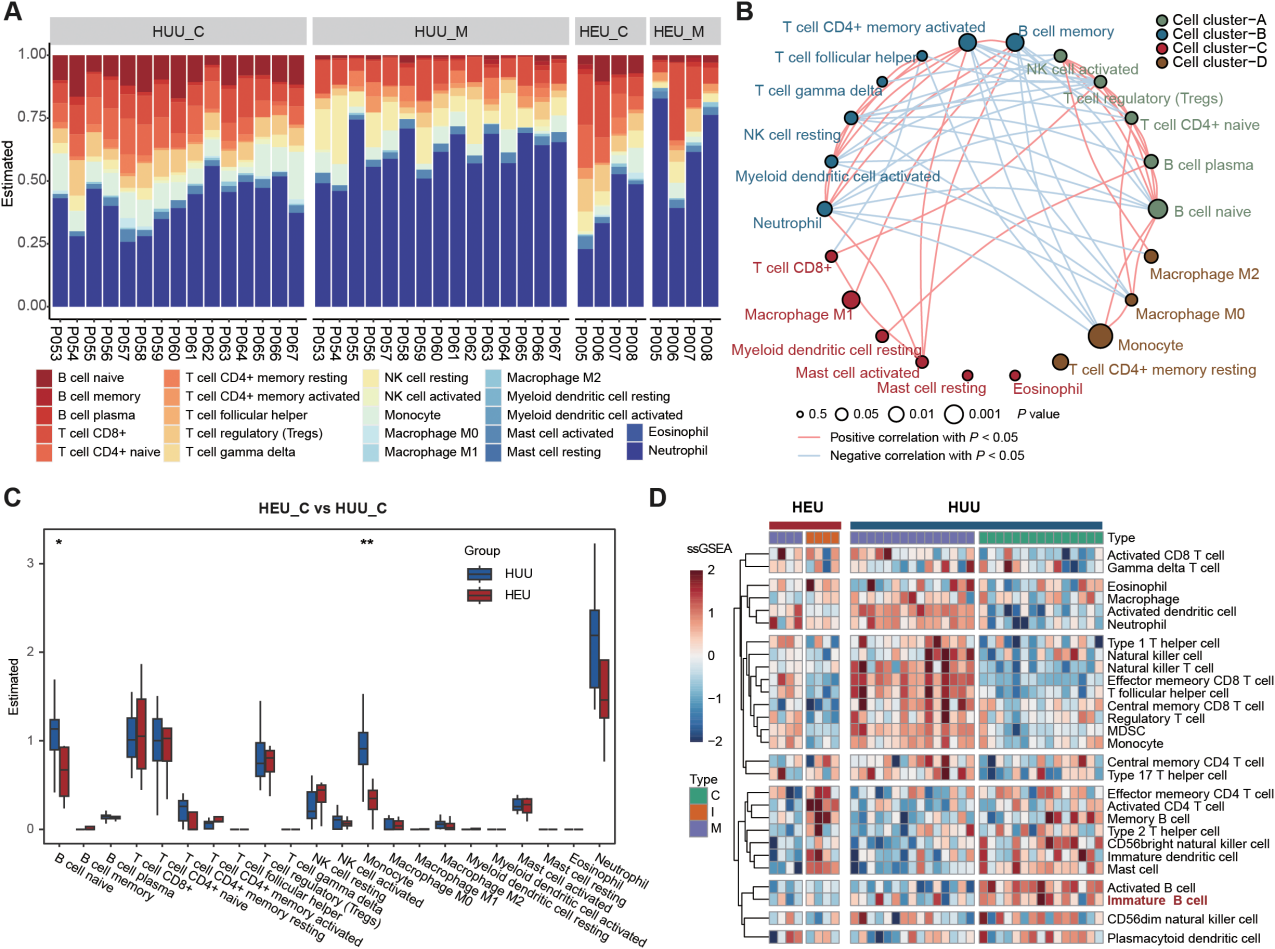
Expression profiling-based immune cell abundance assessment in HEU and HUU subjects. **(A)** Estimation of the abundances of the 22 immune cells using the CIBERSORT algorithm in HEU and HUU subjects. **(B)** Associations between immune cells and cord blood sources. Node size indicates statistical significance by comparing immune cell abundance and B cell levels measured using flow cytometry while line color represents the positive or negative correlation between the abundance among different immune cells. **(C)** Box plots showing differences in the abundance of immune cells in cord blood between HEU and HUU groups. The *P* value was calculated using the Wilcoxon test. *P < 0.05, **P < 0.01. **(D)** Heatmap displaying the different patterns of immune cell composition using ssGSEA (single-sample gene set enrichment analysis) score from different immune cell types.

To further confirm the altered composition of B lymphocyte subsets in cord blood between HEU and HUU, we estimated immune cell abundance based on expression profiling data from all blood samples. Initially, bulk RNA-seq was conducted on 42 blood samples from 19 mother-infant pairs (**Figure 1A**; **Supplementary Table 5**). This technique sequences the RNA in a sample to measure gene expression levels. We than used the CIBSERSORT deconvolution algorithm (24), which infers the proportion of different immune cell types from bulk gene expression data, to identify immune cell composition characteristics (details in **Supplementary Methods**). As shown in **Figure 2A**, distinct immune fractions between HEU and HUU cord blood were revealed, particularly in B lymphocyte subsets.

Moreover, we delved into the underlying relationships among these immune cells and their correlations with HEU cord blood. Given the observed alterations in B cell levels between HEU and HUU cord blood, we evaluated the association with HEU cord blood by comparing immune cell abundance and B cell levels measured using flow cytometry. We observed stronger associations of naive B cells (*P* = 0.0183), monocytes (*P* = 0.0018), and memory B cells (*P* =0.0354) with HEU cord blood. Notably, naive B cells exhibited significant negative and positive correlations with memory B cells (*r* = -0.6414, *P* = 4.725e-06) and monocytes (*r* = 0.3888, *P* = 1.094e-02), respectively (**Figure 2B**). The diminished levels of naive B cells in HEU cord blood were further evidenced by a direct comparison of immune cell abundance in HEU and HUU cord blood (**Figure 2C**). Additionally, we found a significant decrease in monocyte abundance in HEU cord blood, indicating potential abnormalities in the innate immune system of HEU infants.

Furthermore, we conducted a quantitative measurement of immune cell composition via single-sample Gene Set Enrichment Analysis (ssGSEA) (25). This method transforms transcriptomic expression data into normalized scores based on the marker genes of specific immune cell types, providing a comprehensive overview of the immune landscape in each sample (details in **Supplementary Methods**). One of the main advantages of ssGSEA is its ability to provide a consistent measure of immune cell types across different samples, even in heterogeneous data sets. We obtained the relative abundance of 28 immune cell types in all samples with expression data (**Figure 2D**). The result confirmed a significantly lower level of immature B cells in HEU cord blood compared to HUU cord blood (**Supplementary Figure 4**). Altogether, these analyses suggested abnormalities in the function and development of B cells in HEU newborns, possibly related to the abnormal immune system development in early life of HEU infants.

### 3.3 Immune regulatory function of B cells is impaired in HEU infants due to *in-utero* HIV and/or ART exposure

**Figure 3:**
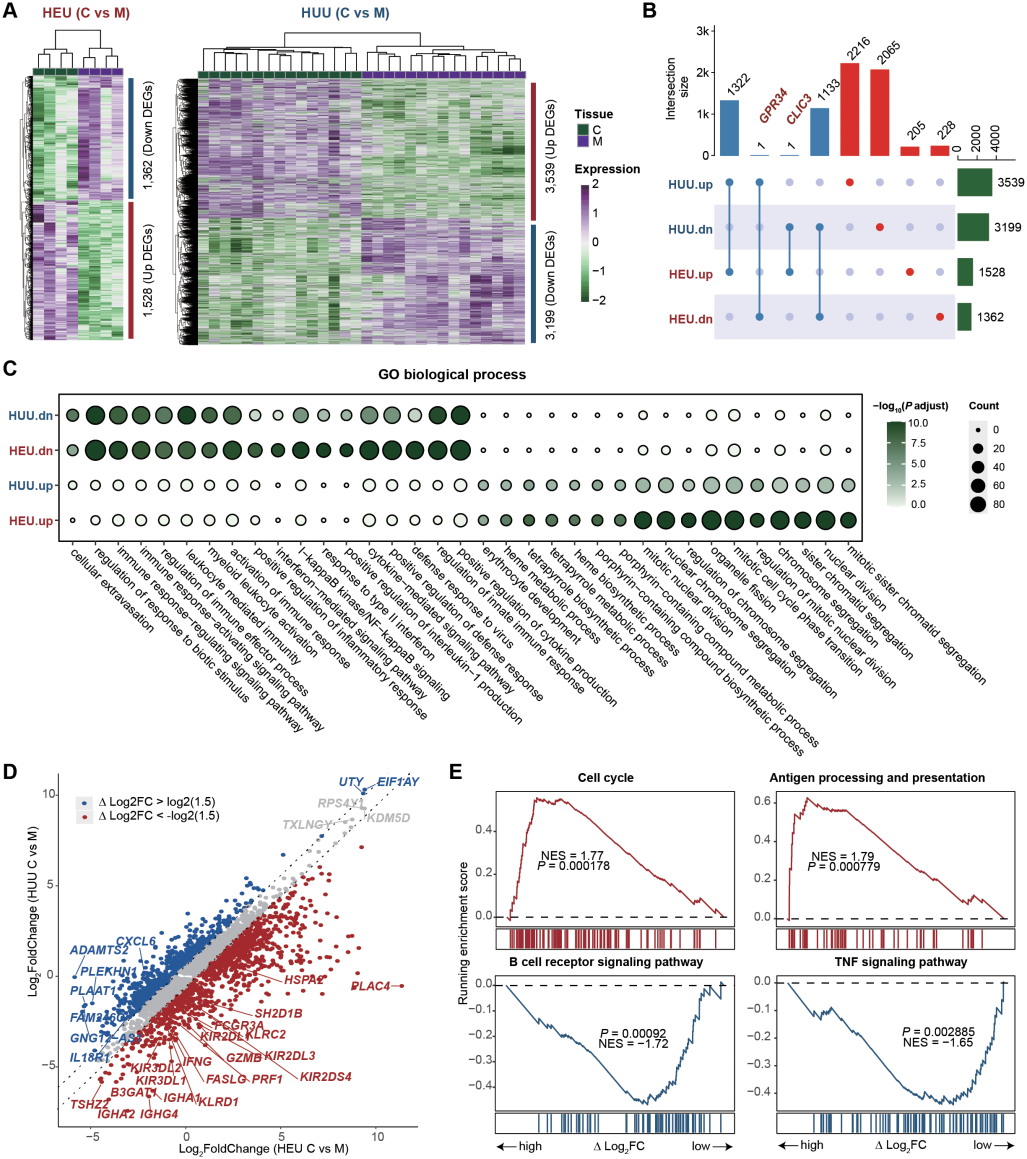
Immune-related pathways are affected in the cord blood of HEU. **(A)** Heatmaps depicting the expression changes of differentially expressed genes (DEGs) between umbilical cord blood and maternal venous blood (C vs M) in HEU (left) and HUU (right) groups, respectively. **(B)** Upset plot illustrating the intersection of the up-regulated and down-regulated DEGs upon C vs M between HEU and HUU. **(C)** GO enrichment analysis showing the biological process terms enriched on the up-regulated and down-regulated DEGs upon C vs M in HEU and HUU. The dot color indicates the *P* value of GO terms calculated by the R package ClusterProfiler, and the dot size indicates the number of genes enriched. **(D)** Scatter plot showing the fold changes of gene expression upon umbilical cord blood versus maternal venous blood (C vs M) in HEU and HUU. The △log_2_FC represents the difference in log_2_-transformed fold changes between HEU and HUU. **(E)** Leading-edge plots showing the enrichment of indicated gene sets. ES, enrichment score; FDR, false discovery rate; scRNA-seq, single-cell RNA-seq. Leading-edge plots showing significant influence on immune-related pathways based on GSEA (red: gene set enriched in HEU samples; blue: gene set enriched in HUU samples). Genes were ranked according to the values of △log_2_FC. NES, normalized enrichment score.

After identifying and characterizing the immune cell composition in HEU cord blood, our focus shifted to comparing the gene expression profiles of HEU and HUU subjects to investigate which immune-related pathways are affected in cord blood (details in **Supplementary Methods**). We found 2,890 genes differentially expressed in HEU and 6,738 in HUU when comparing umbilical cord blood to maternal venous blood (C vs M) (fold change > 1.5; false discovery rate < 0.01) (**Figure 3A**; **Supplementary Tables 6**, **7**). Among these, 206 genes were up-regulated and 229 down-regulated specifically in HEU (**Figure 3B**). Of note, *GRP34* was down-regulated in HEU but up-regulated in HUU, and *CLIC3* was up-regulated in HEU but down-regulated in HUU. The deficiency of *GPR34* and overexpression of *CLIC3* play key roles in altering immune response, inflammatory activation, pathogen susceptibility, and immune infiltration (26–31). Functional enrichment analysis of the top 1000 differentially expressed genes (DEGs) revealed that GO terms related to the innate immune system, such as regulation of response to biotic stimulus and positive regulation of inflammatory response, were consistently enriched in down-regulated DEGs in both HEU and HUU. However, these GO terms were more strongly enriched in HEU, indicating that *in-utero* HIV and/or ART exposure might exacerbate the weakening of the innate immune response in HEU infants (**Figure 3C**).

To understand the specific immunological abnormalities resulting from *in-utero* HIV and/or ART exposure, we conduct a comparative analysis of gene expression fold changes between umbilical cord blood and maternal venous blood (C vs M) in both HEU and HUU groups. We quantized the difference as △log_2_FC: △log_2_FC of *Gene* = Log_2_(FC in HEU C vs M) - Log2(FC in HUU C vs M). We found 1,273 up-regulated genes and 1,306 down-regulated genes in HEU infants due to *in-utero* HIV and/or ART exposure (**Figure 3D**; **Supplementary Table 8**). GSEA was performed by ranking the genes based on △log_2_FC values to elucidate the affected biological pathways. As shown in **Figure 3E**, up-regulated genes were significantly enriched in pathways associated with the activation of immune function in mature B cells, such as Cell cycle and Antigen processing and presentation. Down-regulated genes were notably enriched in the B cell receptor signaling pathway and TNF signaling pathway, suggesting potential inhibition of naïve B cells (**Supplementary Figure 5**). This was consistent with reduced B cell levels in HEU cord blood. These results highlight significant impairment in B cell function in HEU infants, likely due to *in-utero* HIV and/or ART exposure, and underscore the importance of monitoring and potentially adjusting immunization strategies for HEU infants.

### 3.4 WGCNA network module mining identifies HEU-associated co-expression patterns in cord blood

**Figure 4:**
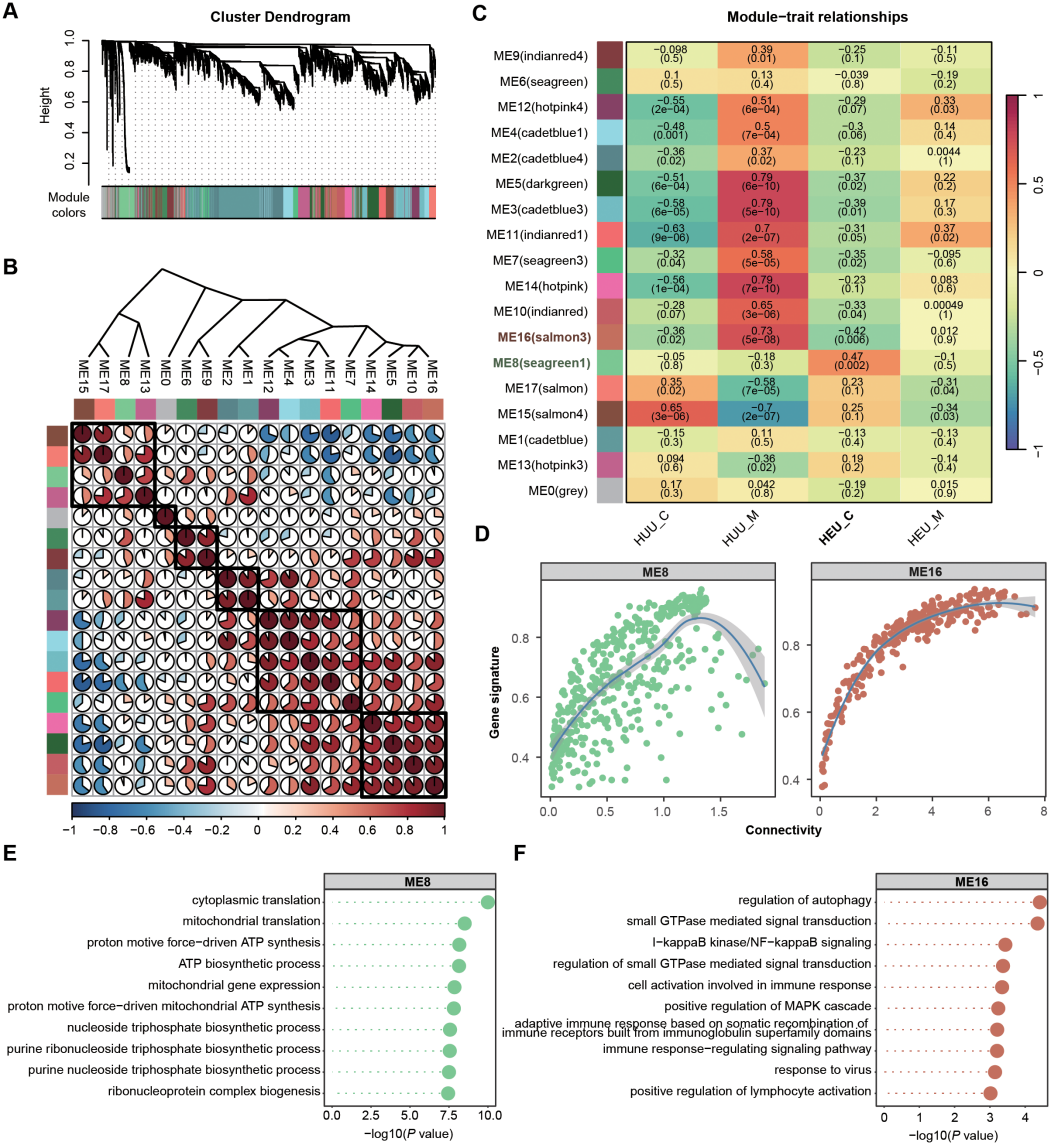
Identification and characterization of cord blood-associated modules in HEU subjects using WGCNA. **(A)** Identification of gene co-expression modules based on the expression profiles using WGCNA. **(B)** Heatmap of eigengene adjacency showing correlations between 17 modules. Positive correlations are indicated in red shades and negative correlations in blue shades. **(C)** Heatmap displaying the correlation between module eigengenes and sample source relationship of 17 modules. The *P* value is indicated in brackets. **(D)** Scatter plots illustrating the intramodular analysis (module membership versus gene significance) of genes in modules (ME8, positive correlation module; ME16, negative correlation module) significantly associated with the cord blood in HEU. **(E, F)** GO enrichment analysis demonstrating the biological process terms associated with the genes in ME8 **(E)** and ME16 **(F)**. *P* value was calculated by the R package ClusterProfiler.

Solely focusing on DEGs in global gene expression profiles may lead to overlooking potentially significant findings. Hence, we employed Weighted Gene Co-expression Network Analysis (WGCNA) to identify HEU-related preserved gene modules in cord blood (details in **Supplementary Methods**). WGCNA is systems biology method used to describe the correlation patterns among genes across RNA-seq samples. It clusters gene into modules based on their co-expression, summarizing each module with its eigengene (the first principal componet) (32). This method allows for the detection of modules are significantly associated with external traits, such as HEU status on our study. We integrated the expression matrices from 42 samples (both cord blood and venous blood) and selected 10,150 genes according to mean and variance criteria (MEAN > 1, VAR > lower quartile) for network construction. WGCNA requires high-quality and high-dimensional data, ensuring sufficient variability to identify meaningful gene modules. In this co-expression network, we identified 17 modules by merging those with eigengene correlations above 0.9 (**Figures 4A, B**). Heatmaps of eigengene adjacency and module-trait relationships revealed that modules ME8 (N = 460) and ME16 (N = 261) were significantly correlated with HEU cord blood (HEU_C), with ME8 being positively and ME16 negatively correlated (**Figure 4C**; **Supplementary Table 9**).

To assess the coherence of gene co-expression within these modules, we calculated the intramodular connectivity, confirming robust internal connectivity for both ME8 and ME16 (**Figure 4D**). Concurrently, we performed gene enrichment analyses based on the GO database to explore the relevant molecular functions of ME8 and ME16. Genes in ME8 were notably enriched in the pathways associated with the activation of mitochondrial function, implying potential abnormalities in cell function homeostasis in HEU cord blood (**Figure 4E**). Nevertheless, genes in ME16 exhibited significant enrichment in the biological processes related to immune responses, such as regulation of autophagy, I−kappaB kinase/NF−kappaB signaling, and positive regulation of MAPK cascade, suggesting suppressed infection-fighting immunity in HEU cord blood (**Figure 4F**). Using WGCNA allowed us to uncover co-expression patterns that might be missed by simply focusing on differentially expressed genes. This comprehensive approach provided deeper insights into the molecular mechanisms affected by *in-utero* HIV and/or ART exposure.

### 3.5 Screening and identification of the immunologically significant HEU-associated signature in cord blood

**Figure 5:**
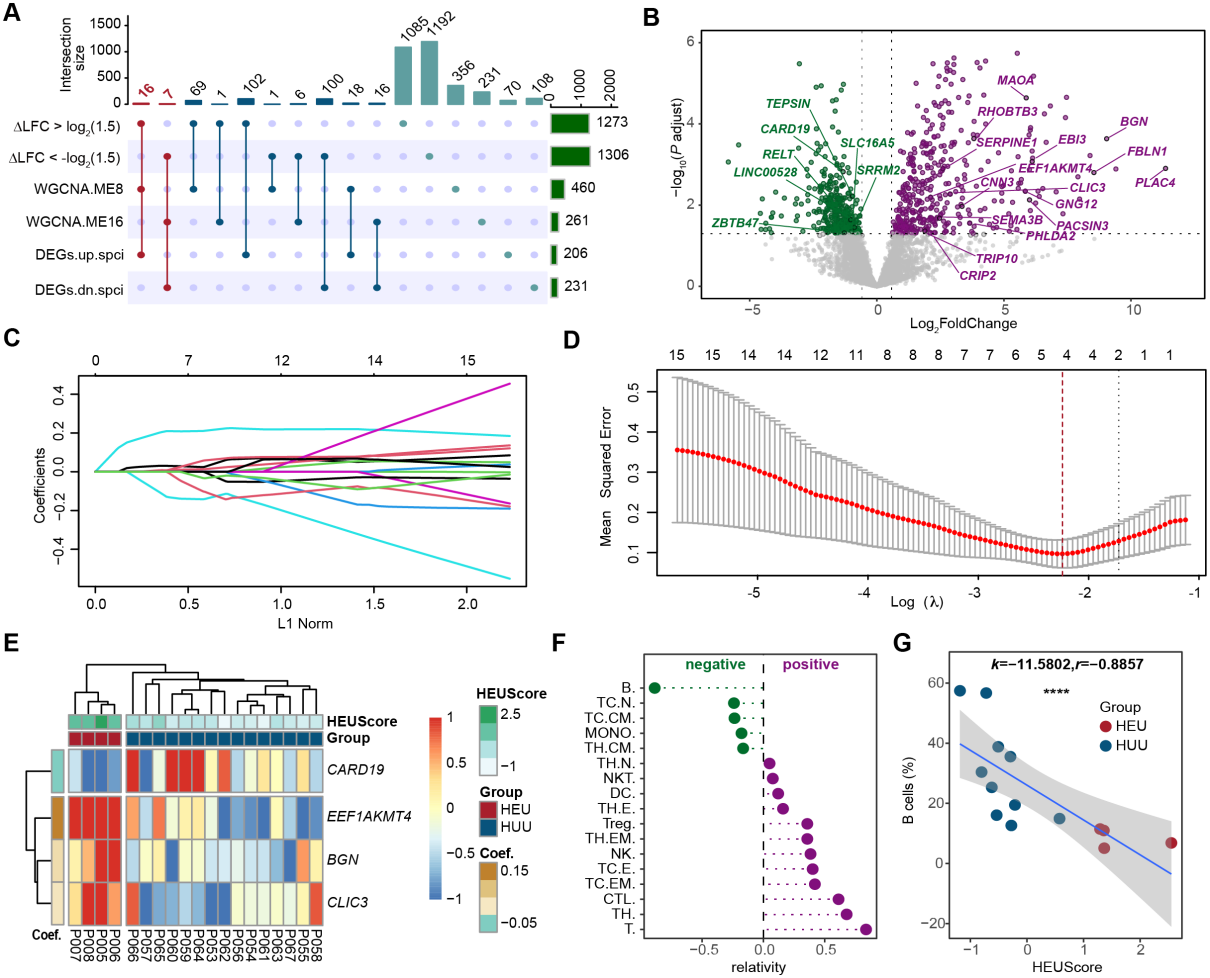
Screening and identification of the pivotal HEU-related signature in cord blood. **(A)** Upset plot displaying the intersection of genes with larger fold change in HEU compared to HUU cord blood (|△log_2_FC| > 1.5), differentially expressed genes specific to HEU cord blood, and genes in the co-expression modules significantly related to HEU cord blood. **(B)** Volcano plot showing the 23 candidate hub genes based on the differential expression results in cord blood versus maternal venous blood datasets from HEU subjects. The dashed line indicates Bonferroni adjusted *P* ≤ 0.05 and ≥ 1.5-fold change. **(C)** Coefficients of each predictor in the LASSO regression model based on the 23 candidate hub genes. **(D)** Identification of 4 hub genes in the pivotal HEU-related signature through LASSO feature selection. **(E)** Heatmap showing expression fold changes of the 4 hub genes in cord blood between HEU and HUU subjects. HEUScore represents the sum of the expression changes of the 4 hub genes multiplied by their LASSO coefficient. **(F)** Correlation analysis between the identified pivotal signature and proportions of different immune cells in cord blood. **(G)** Scatter plot illustrating a strong negative correlation between B cell levels and the identified pivotal signature in cord blood. The x-axis represents the HEUScore, and the y-axis represents the proportion of B cells in the cord blood. *k*, slopes generated by linear regression. *r*, Spearman coefficient. ****P < 0.0001.

To identify the pivotal HEU-related signature and assess its immunological significance, we screened and identified the hub genes by integrating differential expression analysis, co-expression network analysis, and LASSO regression analysis. Initially, we intersected genes with an absolute value of △log_2_FC greater than log_2_(1.5), DEGs specific to HEU cord blood, and genes in co-expression modules ME8 and ME16 (which are significantly associated with HEU cord blood) (**Figure 5A**). This approach led to the identification of 16 up-regulated and 7 down-regulated candidate genes (**Figure 5B**; **Supplementary Table 10**). Next, we employed LASSO (Least Absolute Shrinkage and Selection Operator) regression to refine these candidates. LASSO regression is a statistical method used to identity the most important predictors in a dataset, helping to enhance the model’s interpretability and performance by penalizing the absolute size of regression coefficients. By applying the LASSO model to the 23 candidate genes (**Figure 5C**), we identified 4 hub genes, as indicated by the lowest point on the tenfold cross-validation error curve (**Figure 5D**). These hub genes, namely *CARD19*, *EEF1AKMT4*, *BGN*, and *CLIC3*, constituted a 4-gene HEU-related signature (**Supplementary Figure 6**). We then calculated the HEU-related scores (HEUScore) using the coefficients derived from the LASSO model, formulated as follows: HEUScore = 0.198924 * *EEF1AKMT4* - 0.099502 * *CARD19 +* 0.030197 * *BNG* + 0.006973 * *CLIC3*. Utilizing the expression change matrix of these 4 hub genes, unsupervised clustering successfully distinguished cord blood samples from HEU and HUU, with significantly higher HEUScores observed in HEU cord blood (**Figure 5E**).

Previous findings indicating significant B cell level differences in cord blood between HEU and HUU prompted us to investigate the association between the identified pivotal HEU-related signature and B cell levels. Through flow cytometry-based immunophenotyping and quantization of cord blood immune cell subsets, we evaluated the correlation between various immune cell levels and HEUScore values (**Figure 5F**). Remarkably, we observed a highly significant negative correlation between B cell levels and HEUScore among all immune cell subtypes (*r* = -0.8857, *P* < 2.2e-16) (**Figure 5G**). In summary, our results suggested that the HEU-related signature, composed of the 4 hub genes, bore considerable immunological significance. Furthermore, the HEUScore, calculated based on these genes, effectively assessed B cell levels in cord blood, demonstrating robust predictive efficiency for immune abnormalities in HEU infants.

## 4 Discussion

Previous studies have underscored the heightened infectious morbidity and mortality among HEU infants compared to HUU infants, highlighting the pressing need for mechanistic insights into the immune dysfunction induced by *in-utero* HIV and/or ART exposure. In this study, we embarked on a systematic exploration of the altered immune system status in HEU infants at birth, leveraging both immunophenotypic data and RNA-seq data from mother-infant pairs. We found a significant reduction in B lymphocyte subsets in HEU cord blood, along with suppressed innate immune responses and affected B cell regulatory functions. Additionally, we identified an HEU-related signature capable of assessing B cell levels in cord blood to discern between HEU and HUU infants, providing novel biomarkers for immunological abnormalities in HEU infants.

Our extensive comparison of immunophenotypes in umbilical cord blood and maternal venous blood unveiled several immunological distinctions between HEU and HUU cohorts. Elevated systemic inflammation and immune activation in HIV+ mothers have been implicated in adverse infant clinical outcomes (9, 33). As expected, we observed altered immune cell compositions in maternal venous blood, with increased T cells, dendritic cells, and monocytes (34), and a decrease in NK cells, potentially linked to impaired CD4 recovery post-treatment (35). Intriguingly, our data revealed a noteworthy finding: significantly lower levels of B lymphocyte subsets in HEU cord blood compared to HUU, representing the sole statistically significant change in immune cell composition observed. This may elucidate the negative impact of maternal HIV infection on passive immune transfer to infants during pregnancy (36). In addition, several studies have reported monocyte activation in HEU infants (19, 22, 37, 38), which deserves further verification in our follow-up data. In brief, the above-mentioned changes in immune phenotypes in cord blood corroborate altered immune development in HEU infants.

We compared functional analysis results of HEU and HUU to understand the unique effects of *in-utero* HIV and/or ART exposure. Previous reports indicate that *in-utero* HIV and/or ART exposure can alter the innate immune response in HEU infants due to heightened inflammation and immune activation (20, 39–41). Our findings reflect this, showing that while GO results were similar between the HEU and HUU groups, HEU exhibited greater enrichment in innate immune response-related pathways among down-regulated DEGs. Integrating the differential expression results revealed abnormalities in B cell function in HUE. Specifically, we observed both activation of antigen presentation pathways and inhibition of the BCR signaling pathways. Key signaling subunits Igα(CD79a) and Igβ(CD79b) are vital for initiating BCR signal transduction (42–44), and minimal changes in the expression levels of their encoding genes (*CD79A* and *CD79B*) in the HEU group underscore impairments in B cell development and function. Despite these observed B cell abnormalities, HEU infants exhibit an adequate humoral immune response to primary vaccination (16, 45–47). This effectiveness can be attributed to the activation of antigen processing and presentation pathways, which are crucial for promoting the humoral response development (48). Thus, we infer that while HEU newborns display defective B-cell function and development, their adequate vaccine response primarily due to maternal transfer of antimicrobial immunity.

Our study aimed to identify biomarkers in cord blood influencing immune development in HUE infants using comprehensive expression data analysis. Through WGCNA, we found that modules positively associated with HEU cord blood indicated mitochondrial dysfunction, aligning with studies linking altered mitochondrial metabolism and poor clinical outcomes in HEU infants (49–52). Modules negatively associated with HEU cord blood supported the notion of suppressed immune system activity in HEU infants. From the co-expression network and differential expression analysis, we derived a four-gene HEU-related signature using the LASSO algorithm. Notably, the HEUScore derived from this signature was tightly associated with the B cell levels in cord blood. Interestingly, *CLIC3*, a key gene in this signature, was specifically overexpressed in HEU cord blood. *CLIC3* is known to activated the NLRP3 inflammasome, which can sense different pathogens or danger signals (31), and its overexpression has beeb linked to immune evasion in cancer cells (53). Importantly, the 4-gene signature, previously unreported, holds promise for predicting immunological abnormalities in HEU cord blood, potentially facilitating clinical implementation. Admittedly, the causal relationship between this identified signature and adverse clinical outcomes in HEU infants warrants further validation.

This study represents the first comprehensive immunological and transcriptomic characterization of HEU cord blood, resulting in a four-gene-based classifier that holds potential for predict immunological abnormalities in HEU infants. Our findings delineate abnormalities in B cell composition and function in HEU cord blood, opening avenues for further investigation. It is deducible that altered B lymphocyte function caused by *in-utero* HIV and/or ART exposure likely contributes to immune dysregulation development and subsequent susceptibility to infectious diseases. These insights underscore the importance of follow-up studies to monitor immune dynamics and infection status in HEU infants, with future research focusing on longitudinal investigations to further elucidate these relationships.

## Data Availability

'The data presented in the study are deposited in the National Omics Data Encyclopedia (NODE) repository, accession number OEP005165.
