# Supplementary material for: Reduced B cell frequencies in cord blood of HIV-exposed uninfected infants: an immunological and transcriptomic analysis
Source: Front Immunol. 2024 Sep 4;15:1445239. doi: 10.3389/fimmu.2024.1445239 (PMC11408218; doi:10.3389/fimmu.2024.1445239)
Supplement: Supplementary file 1 [file DataSheet1.pdf]

# *Supplementary Material*

**1 Supplemental Methods…………………………………………………….............2**

**2 Supplemental Figures......……………………………………………………....7**

**3 Supplemental References………………………………………………………12**

# 1 Supplemental Methods

## 1.1 Immunological Profiling

A total of 56 blood samples, comprising 9 HEU mother-child pairs and 19 HUU mother-child pairs, were successfully analyzed to construct immune profiles using flow cytometry. These profiles included the composition of 17 immune cell subsets, encompassing mononuclear cells (MONO), dendritic cells (DC), T cells, B cells, and natural killer cells (NK). T cell subsets were categorized into T helper cells (TH), cytotoxic T cell (CTL) and natural killer T cells (NKT), of which TH was subdivided into regulatory T cell (Treg), naïve CD4+ T cells (TH-N), central memory CD4+ T cells (TH-CM), effector CD4+ T cells (TH-E) and effector memory CD4+ T cells (TH-EM), and CTL was subdivided into naïve CD8+ T cells (TC-N), central memory CD8+ T cells (TC-CM), effector CD8+ T cells (TC-E) and effector memory CD8+ T cells (TC-EM) .

Antibody expression across these 17 immune cell types was summarized in supplement Table 4. Flow cytometry was employed to determine the relative abundance of these cell subsets, providing a comprehensive overview of the immunological landscape in HEU and HUU subjects. The analysis involved staining the blood samples with a panel of fluorochrome-conjugated antibodies (supplement Table 3) specific to surface markers characteristic of the immune cell subsets mentioned. Data acquisition was performed using a flow cytometer, and subsequent data analysis was conducted using specialized software to gate and quantify the populations of interest (supplement Figure 2).

To assess differences in cell type composition between HEU and HUU groups, we employed the Wilcoxon rank-sum test. This non-parametric test was chosen due to its robustness in handling small sample sizes and non-normal distributions, making it suitable for the comparative analysis of immune cell frequencies. Differences were considered statistically significant at a $P$ value $< 0.05$. This statistical approach enabled us to identify specific immune cell subsets that were differentially represented between HEU and HUU cohorts.

## 1.2 RNA-seq Data Processing

Bulk RNA-seq was conducted on 38 blood samples from 4 HEU mother-child pairs and 15 HUU mother-child pairs (supplement Table 5). The RNA-seq data processing workflow involved several key steps to ensure accurate alignment, quantification, and normalization of the transcriptomic data. Raw sequencing reads first quality-checked and trimmed to remove adapters and low-quality bases using fastp (0.23.2)(1). The high-quality were then aligned to the reference human genome hg38 (Genome Reference Consortium GRCh38) by Hisat2 (v2.2.1)(2). Following alignment, the gene counts of each sample were generated using featureCounts (v2.0.1)(3). To facilitate comparison between samples, gene counts were normalized to transcripts per million (TPM). TPM normalization accounts for both the sequencing depth and the gene length, allowing for the direct comparison of gene expression levels across different samples. This dataset was subsequently used for downstream analyses, including differential expression analysis, gene ontology (GO) enrichment analysis, and gene set enrichment analysis (GSEA).

## 1.3 Estimation of Immune Cell Abundance

Normalized gene expression profiles were employed to estimate immune cell abundance. CIBERSORT (https://cibersort.stanford.edu/) was utilized to determine the proportion of immune cells based on the LM22 gene signature(4). Meanwhile, quantitative assessment of immune cell composition was carried out using single-sample Gene Set Enrichment Analysis (ssGSEA) based on the marker genes representing 28 specific immune cell types(5), implemented via the R package GSVA(6). CIBERSORT and ssGSEA complement each other in analyzing immune cell proportions by combining the precise cell type identification and individual-level analysis of CIBERSORT with the flexibility and broad applicability of ssGSEA. While CIBERSORT provides accurate estimates of immune cell proportions using reference gene expression profiles, ssGSEA offers relative abundance information without needing predefined reference profiles and is robust across various data types. By integrating the precise quantification from CIBERSORT with the functional enrichment insights from ssGSEA, we can achieve a more comprehensive understanding of immune cell composition and functionality.

### 1.4 Differential Expression Analysis

Gene count files were utilized for conducting differential expression analysis between umbilical cord blood and maternal venous blood samples from both HEU and HUU subjects. This analysis was carried out using the R package DESeq2 (v1.39.8), which employs a model based on the negative binomial distribution(7). DESeq2 normalizes the data and estimates the size factors to control for differences in sequencing depth and RNA composition between samples. Genes exhibiting a $\log_2$ fold change ($\log_2$FC) greater than or equal to $\log_2(1.5)$ and a $P$ value < 0.05 were considered differentially expressed. The $\log_2$FC threshold of 1.5 corresponds to a 1.5-fold change in gene expression, which is generally considered meaningful in the context of gene regulation. The $P$ value threshold of 0.05 controls the false discovery rate, ensuring that the results are reliable.

### 1.5 Functional Enrichment Analysis

Gene Ontology (GO) enrichment analysis and gene set enrichment analysis (GSEA) based on the Kyoto Encyclopedia of Genes and Genomes (KEGG) were conducted using the R package clusterProfiler (v4.9.0)(8). The enrichGO function in clusterProfiler was employed, using a hypergeometric test to determine the overrepresentation of specific GO terms among the DEGs compared to what would be expected by chance. Adjustments for multiple testing were made using the Benjamini-Hochberg procedure to control the false discovery rate (FDR), with significantly enriched GO terms identified at an adjusted $P$ value threshold of <0.05. The gseKEGG function in clusterProfiler was used to perform GSEA, calculating an enrichment score (ES) for each gene set to reflect the degree of overrepresentation at the top or bottom of the ranked gene list. The statistical significance of the ES was assessed using a permutation test, with gene sets considered significantly enriched at an adjusted $P$ value <0.05 and an FDR <0.25. This approach identified key KEGG pathways that exhibit coordinated differences between HEU and HUU groups.

### 1.6 Weighted Gene Co-expression Network Analysis

Weighted gene co-expression network analysis (WGCNA) was employed to identify co-expression modules using the R package WGCNA (v1.72-1)(9). The analysis

involved several steps to ensure the robustness and relevance of the detected co-expression modules. Firstly, the expression matrices were normalized to transcripts per million (TPM) to facilitate comparison between samples. We selected 10,150 genes for network construction according to mean and variance criteria (MEAN > 1, VAR > lower quartile). These criteria ensured the inclusion of genes with sufficient expression levels and variability, which are essential for reliable network construction.

To build the co-expression network, we applied WGCNA, setting the soft threshold power to 18. This threshold was chosen because it achieved a scale independence reached 0.76 and the average connection value of 30.53, which are indicative of a robust network. The sensitivity for module detection was set a medium level of 2.0, ensuring a balance between detecting distinct modules and maintaining module robustness. The cut height for merging modules was set to 0.1, allowing closely related modules to be combined, thereby reducing redundancy. Additionally, we set the minimum module size to 150 to focus on biologically significant modules while filtering out smaller, potentially less relevant ones.

Finally, modules whose eigengenes (the first principal component of the module expression data) were correlated above 0.9 were merged. This step ensured that highly similar modules were consolidated, enhancing the biological interpretability and stability of the identified co-expression modules. This comprehensive approach allowed us to detect robust co-expression modules, which were further analyzed to uncover their biological significance and potential roles in the immunological differences observed between HEU and HUU subjects.

## 2 Supplemental Figures

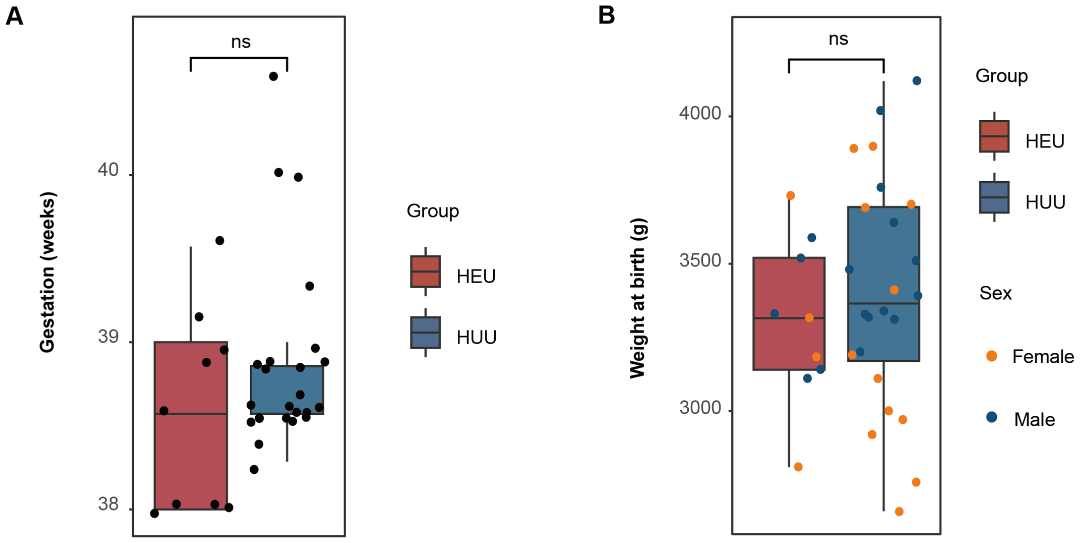


**Supplemental Figure 1. Clinical statistics between HEU and HUU groups.**

(A) Comparison of gestational time between HEU and HUU groups. (B) Comparison of infant birth weight between HEU and HUU groups. The colors of the box dots represent different genders.

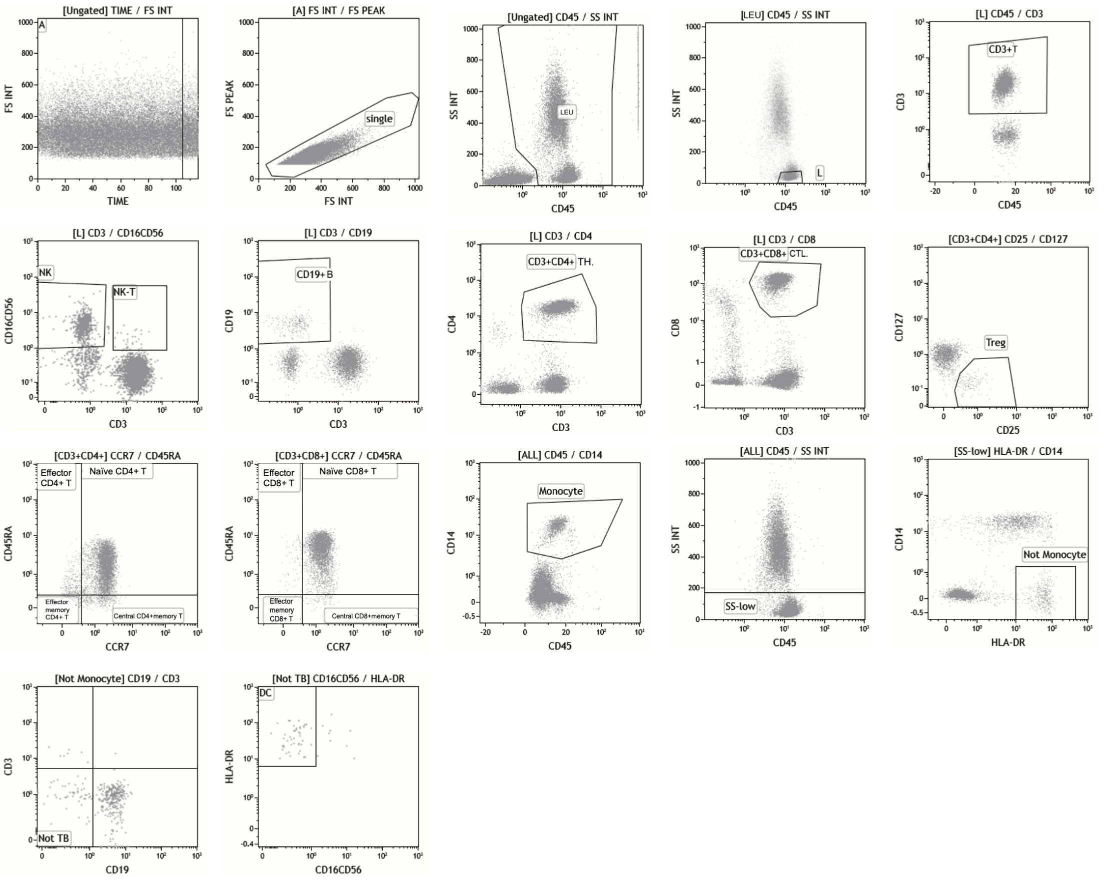


**Supplemental Figure 2. Flow cytometry gating strategy.**

This figure illustrates the gating strategy applied to the representative cord blood sample from subject P014. Initially, a time gate was used to exclude electronic noise, followed by a size gate to select single cells. For lymphocytes, gating was performed using SSC and CD45. Major T-cell populations were identified using CD3, CD4, and CD8 markers, with CD45RA and CD197 (CCR7) combinations used to distinguish naïve, central memory, effector, and effector memory subpopulations. NK and NKT cells were distinguished using CD3, CD16, and CD56 markers. CD19 and CD14 markers were used to identify B cells and mononuclear cells, respectively. Dendritic cells (DCs) were identified as CD3-CD14-CD56-CD19-HLA-DR+.

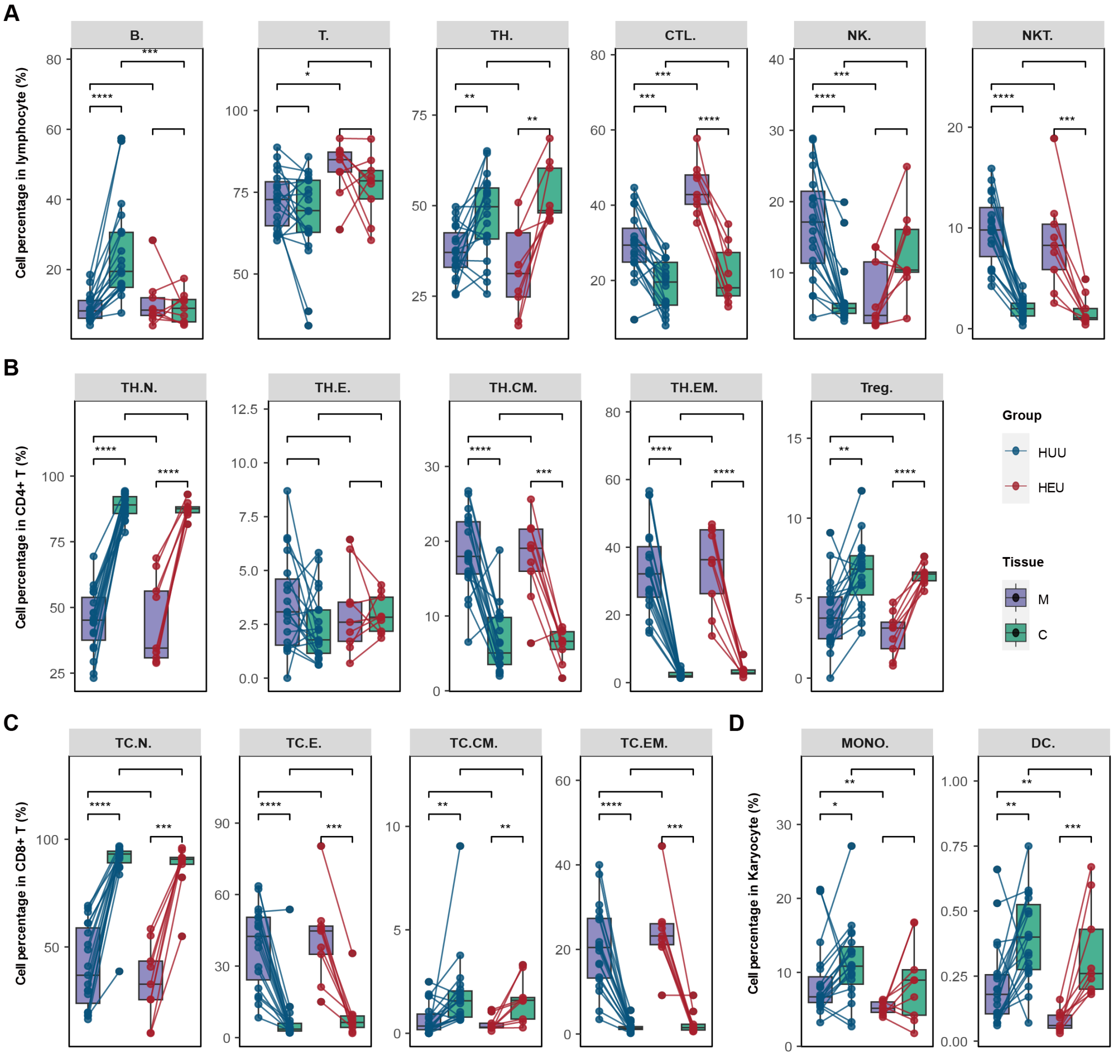


**Supplemental Figure 3. Matched analysis of the compositions of various immune cells in maternal venous blood and umbilical cord blood samples across the HEU and HUU subjects.**

(A) Frequency of immune cell types as a percentage of all lymphocytes (gated on CD45 vs. SSC). (B) Frequency of immune cell types as a percentage of all CD4+ T cells (gated on CD3 vs. CD4). (C) Frequency of immune cell types as a percentage of all CD8+ T cells (gated on CD3 vs. CD8). (D) Frequency of immune cell types as a percentage of all karyocytes. Each box plot represents the median, interquartile range, and minimum and maximum quartile of expression. The *P* value was calculated by the Wilcoxon test. **P* < 0.05, ***P* < 0.01, ****P* < 0.001, *****P* < 0.0001.

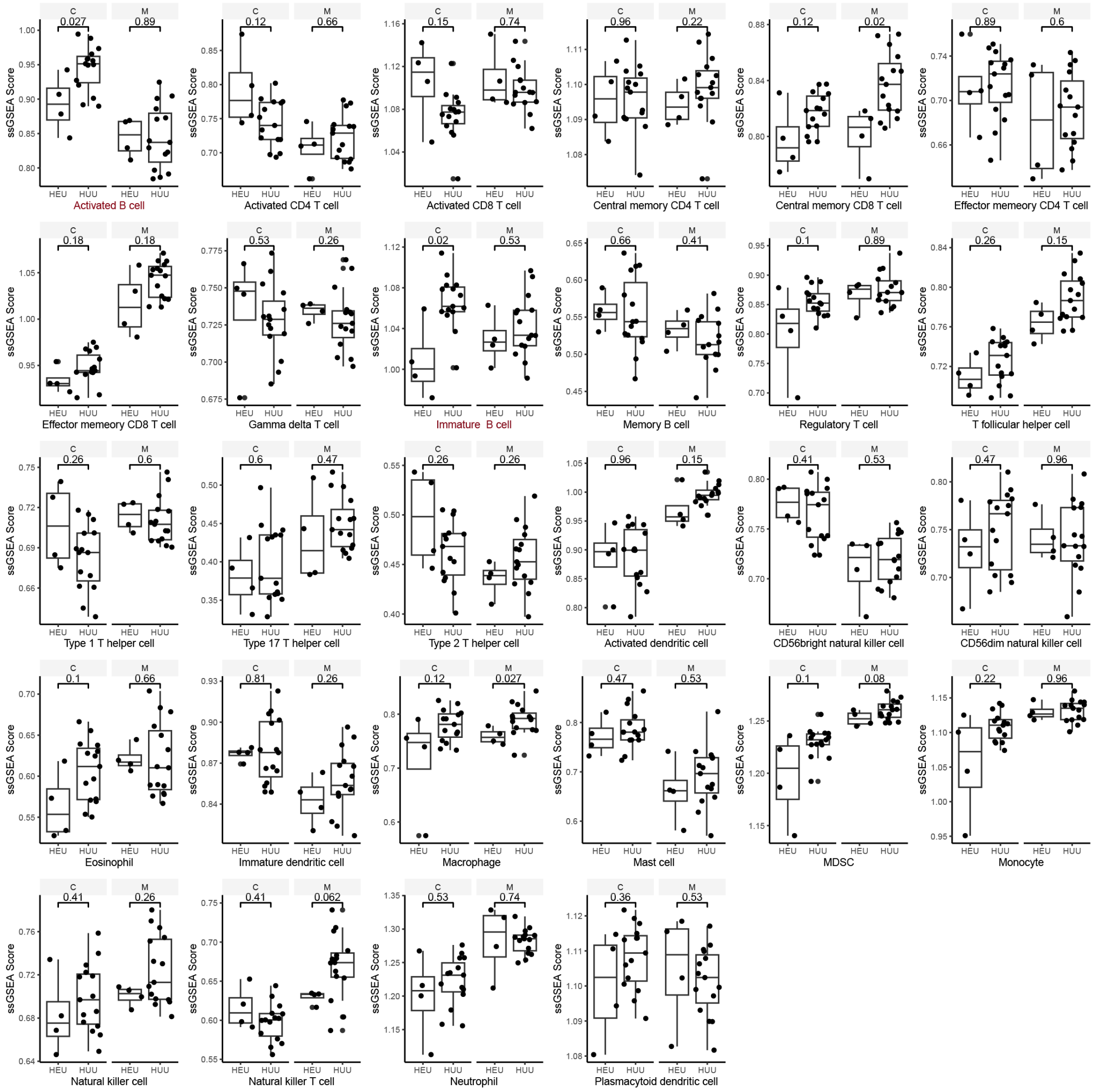

**Supplemental Figure 4. Comparison of the different patterns of immune cell composition using ssGSEA.**

Each box plot represents the median, interquartile range, and minimum and maximum quartile of expression. The *P* value was calculated by the Wilcoxon test, with statistical significance defined as *P* < 0.05.

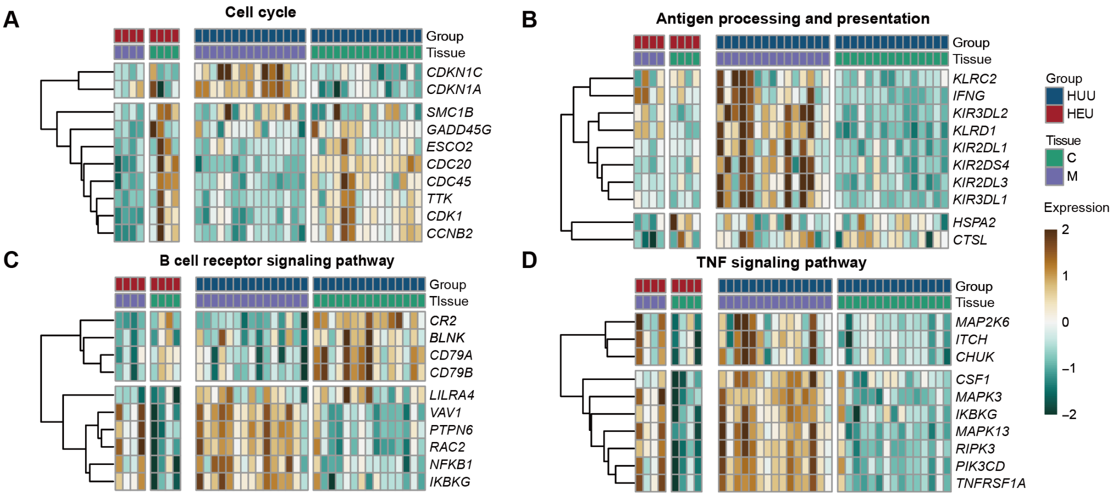


**Supplemental Figure 5. Heatmaps showing the expression of the top 10 core genes in the affected immune-related pathways across different tissue samples in HEU and HUU.**

GSEA was performed by ranking the genes based on $\Delta \log_2 FC$ values to elucidate the affected biological pathways. up-regulated genes were significantly enriched in the pathways associated with the activation of immune function in mature B cells, such as Cell cycle (A) and Antigen processing and presentation (B). Down-regulated genes were notably enriched in the B cell receptor signaling pathway (C) and TNF signaling pathway (D).

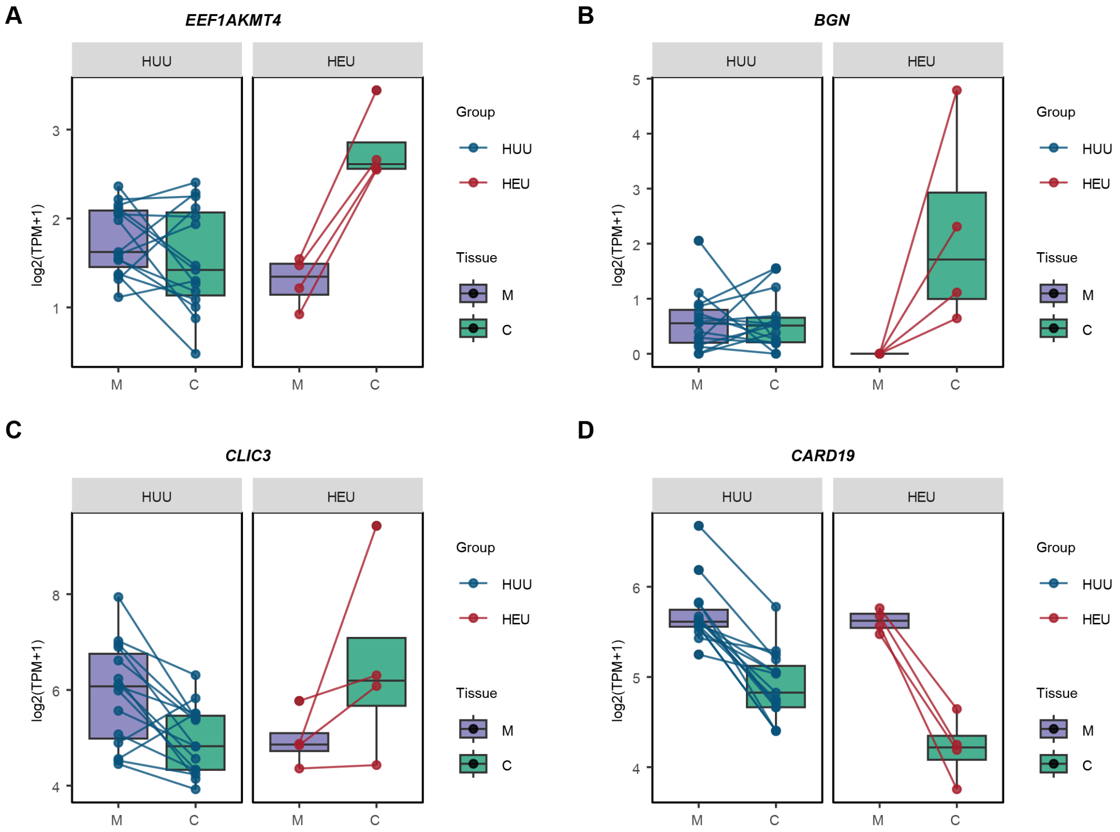


**Supplemental Figure 6. Expression trends for the identification of four hub genes that constitute the pivotal HEU-related signature.**

The hub genes including *EEF1AKMT4* (A), *BGN* (B), *CLIC3* (C) *and CARD19* (D). Each box plot represents the median, interquartile range, and minimum and maximum quartile of expression.

## 3 Supplemental References

1. Chen S. Ultrafast one-pass FASTQ data preprocessing, quality control, and deduplication using fastp. Imeta. 2023;2(2):e107.

2. Kim D, Paggi JM, Park C, Bennett C, Salzberg SL. Graph-based genome alignment and genotyping with HISAT2 and HISAT-genotype. Nat Biotechnol. 2019;37(8):907-15.

3. Liao Y, Smyth GK, Shi W. featureCounts: an efficient general purpose program for assigning sequence reads to genomic features. Bioinformatics. 2014;30(7):923-30.

4. Newman AM, Steen CB, Liu CL, Gentles AJ, Chaudhuri AA, Scherer F, et al. Determining cell type abundance and expression from bulk tissues with digital cytometry. Nat Biotechnol. 2019;37(7):773-82.

5. Charoentong P, Finotello F, Angelova M, Mayer C, Efremova M, Rieder D, et al. Pan-cancer Immunogenomic Analyses Reveal Genotype-Immunophenotype Relationships and Predictors of Response to Checkpoint Blockade. Cell Rep. 2017;18(1):248-62.

6. Hanzelmann S, Castelo R, Guinney J. GSVA: gene set variation analysis for microarray and RNA-seq data. BMC Bioinformatics. 2013;14:7.

7. Love MI, Huber W, Anders S. Moderated estimation of fold change and dispersion for RNA-seq data with DESeq2. Genome Biol. 2014;15(12):550.

8. Wu T, Hu E, Xu S, Chen M, Guo P, Dai Z, et al. clusterProfiler 4.0: A universal enrichment tool for interpreting omics data. Innovation (Camb). 2021;2(3):100141.

9. Langfelder P, Horvath S. WGCNA: an R package for weighted correlation network analysis. BMC Bioinformatics. 2008;9:559.
